# Memristive ternary Łukasiewicz logic based on reading-based ratioed resistive states (3R)

**DOI:** 10.1098/rsta.2023.0397

**Published:** 2025-01-16

**Authors:** Feng Liu, Leon Brackmann, Xianyue Zhao, Nan Du, Rainer Waser, Stephan Menzel

**Affiliations:** ^1^ Peter Gruenberg Institut (PGI-7), Forschungszentrum Juelich GmbH, Juelich, Germany; ^2^ Institute of Electronic Materials II, RWTH Aachen University, Aachen, Germany; ^3^ Institute for Solid State Physics, Friedrich Schiller University Jena, Jena, Germany; ^4^ Department of Quantum Detection, Leibniz Institute of Photonic Technology, Jena, Germany

**Keywords:** ternary logic, computation in memory (CIM), RRam

## Abstract

The thirst for more efficient computational paradigms has reignited interest in computation in memory (CIM), a burgeoning topic that pivots on the strengths of more versatile logic systems. Surging ahead in this innovative milieu, multi-valued logic systems have been identified as possessing the potential to amplify storage density and computation efficacy. Notably, ternary logic has attracted widespread research owing to its relatively lower computational and storage complexity, offering a promising alternative to the traditional binary logic computation. This study provides insight into the feasibility of ternary logic in the CIM domain using resistive random-access memory (ReRAM) devices. Its multi-level programming capability making it an ideal conduit for the integration of ternary logic. We focus on ternary Łukasiewicz logic because its computational characteristics are highly suitable for mapping logic values with input and output signals. This approach is characterized by voltage-reading-based output for ease of subsequent utilization and computation and validated in 1T1R crossbar arrays in an integrated ReRAM chip (Memory Advanced Demonstrator 200 mm). In addition, the effect of variability of memristive devices on logical computation and the potential for parallel operation are also investigated.

This article is part of the theme issue ‘Emerging technologies for future secure computing platforms’.

## 1. Introduction

In the contemporary information era, the exponential growth of data volume has imposed significant demands on the speed and efficiency of data processing [1,2]. The bottleneck challenge inherent in conventional von Neumann architecture has progressively hindered its ability to match the escalating demands for data processing speed, primarily owing to constraints in data transfer between processing units and memory. To address this bottleneck, the concept of computation in memory (CIM) has emerged [3]. It has been demonstrated that memristive devices, characterized by their capability to adjust resistance through the modulation of current flow, represent promising candidates for CIM applications. Consequently, there has been a surge in research interest directed towards realizing logical functionalities within memristive devices. Borghetti *et al*. introduced a stateful binary logic operation for material implication employing two memristive devices as a pioneering endeavour in this domain [4]. Exploiting the voltage division effect of two series-connected memristive devices, Kvatinsky *et al.* introduced memristor ratioed logic [5]. They also utilized a circuit configuration comprising three memristive devices to devise a stateful memristor-aided logic gate [6]. In [7], a novel CMOS-like gate is introduced, wherein traditional pMOS and nMOS transistors are substituted with anti-polarized memristive devices. Gao *et al.* combined CMOS and memristive devices to implement a universal threshold logic gate [8]. By encoding inputs as voltage drops across a single memristive device further non-stateful binary imply logic concepts have been proposed, and by combining the devices in a complementary structure other logic function such as NOR and AND can also be implemented [9]. Subsequent studies [10–12] have demonstrated more intricate logical functions such as adders and multipliers. However, to enhance data processing capabilities, multi-valued logic remains an area of sustained interest. The efficiency of a three-valued numbering system is maximized when its bases are integers [13]. The absence of true multi-state devices has historically hindered the adoption of multi-valued computation. In the contemporary landscape, the multi-resistive states exhibited by memristive devices have significantly propelled the advancement of multi-valued logic, thereby enhancing both data storage density and computational efficiency. Utilizing TaO-based memristive devices founded on the valence change mechanism, Kim *et al.* demonstrated 3-bit multi-level switching in the gradual RESET process [14]. Luo *et al.* proposed a novel memristor-based ternary logic design capable of performing stateful ternary logic operations such as AND, OR and NOT using three memristive devices and two switches [15]. A ternary memristor-CMOS logic family has also been experimentally demonstrated by Wang *et al.* [16]. Fey *et al.* proposed an adder in the balanced ternary weighted numbering system [17], as well as a carry-free ternary adder [18]. However, all the aforementioned ternary logic concepts rely upon memristive device switching, thereby leading to increased energy consumption, imposing significant demands on the device endurance and facing highly challenging switching variability of devices. Depending on the operating environment and driving control conditions, resistive random-access memory (ReRAM) devices typically endure between $10^{5}$ and $10^{8}$ cycles [19,20]. To reduce the variability of memristive devices, Nikam *et al.* demonstrate that incorporating a defective graphene monolayer in the threshold-switching (TS) device significantly reduces variability and enhances reliability by regulating the conductive filament formation process, albeit with an increase in threshold voltage [21]. To reduce the demands on device endurance and mitigate the negative effect of variability during the switching process, we propose a novel logic concept for ternary Łukasiewicz logic, based on ratioed resistive states (3R) of two serially connected memristive devices [22] and have validated the concept in this study. Key points are listed below:

— We experimentally validated the proposed method for implementing IMP logic operations based on Łukasiewicz ternary logic in 1T1R crossbar arrays using the commercial Memory Advanced Demonstrator 200 mm (MAD200) chip.
— This method utilizes resistance states of ReRAM devices as inputs and voltage signals as outputs, relying solely on read operations, thereby completely avoiding the switching behaviour of the device during computation.
— Although independent of device switching behaviour, we investigated the effect of memristor variability on the final logic operations, including device-to-device (D2D), cycle-to-cycle (C2C) and read-to-read (R2R) variability during the read/write processes.
— We demonstrated the feasibility/potential of the parallel computation mode through comparative experiments.

## 2. Ternary Łukasiewicz logic based on 3R

**Figure 1.**
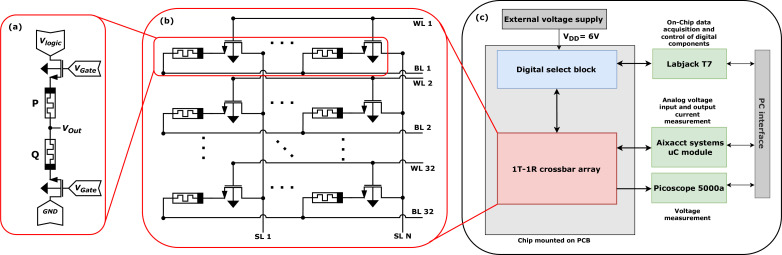
Schematic representation of (*a*) the individual logic unit cell consisting of two anti-serial connected 1T1R devices, (*b*) the crossbar array as part of the integrated ReRAM chip and (*c*) the test set-up used for electrical measurement.


Ternary Łukasiewicz logic, named after Jan Łukasiewicz, is a formal system within mathematical logic that extends classical Boolean logic to accommodate three values: false, indeterminate and true. Conventionally, false is assigned to 0, indeterminate is mapped to 1/2 and true is associated with the value of 1 (asymmetrical ternary system) [23]. Ternary Łukasiewicz logic is characterized by its algebraic structure and logical operators, which include conjunction (AND), disjunction (OR), negation (NOT) and implication (IMP). Furthermore, AND, OR, NOT and other Boolean logic functions can be represented by the combination of IMP and false (0) [24]. For example, NOT is indicated in the IMP operation: $F_{⌝}(p)=p\;\text{IMP}\;0$ and NAND function can be computed in $F_{NAND}(p,q)=p\;\text{IMP}\;(q\;\text{IMP}\;0)$ . In contrast to the original ternary Łukasiewicz logic, we employ the substitution of 2 in lieu of 1 and 1 in lieu of 1/2 to denote logical values. The resultant truth table for the IMP operation is listed in the left-hand side of the accompanying table 1. Its mathematical characterization is as follows: $F_{\to }(p,q)=\text{min}\{2,2-p+q\}$ . From the truth table, it is readily apparent that along the diagonal line from the top left to the bottom right, the output logic values are equivalent. Simultaneously, the depiction of such a diagonal line also implies that the differences between the corresponding inputs p and q , are also equal, which means: if p−q=x or q−p=x , then $F_{\to }(p,q)=y$ , where x indicates the difference of input logic values while y represents the output logic values which can ultimately be mapped to the output signals. Another point to note is that all diagonals (with direction from the top left to the bottom right) from the middle to the top right corner represent the output being the same logic value, describing the characteristic: if q≥p , then $F_{\to }(p,q)=z$ (in this scenario z=2 ). These characteristics are highly suitable for implementing ternary Łukasiewicz implication logic using memristive devices. The mathematical subtraction p−q can be mapped to the voltage drop between the two terminals of the memristive device in a circuit. The resistive states of the devices are delivered as outputs [25,26], listed on the right-hand side of table 1. These characteristics also inspired our concept using the voltage division between two (anti-) serially connected memristive devices as a reading-based ternary IMPLY gate. The resistive states of the two serially connected memristive devices function as logical inputs, represented as P and Q . Each logical input has three resistance states. With the voltage divider effect, an output voltage $V_{\text{out}}$ at the middle node should deliver the expectation of the IMP function when applying a reading voltage $V_{\text{logic}}$ [22] (cf. figure 1*a*). With the voltage divider effect, it is easy to obtain

**Table 1.** IMP truth table (left) and the mapping of logic values to the information in circuits (right).

*p*\*q*	0	1	2	q−p	−2	−1	0	1	2
0	2	2	2	logic value	0	1	2	2	2
1	1	2	2	* $V_{out}$* [V]	0.06	0.20	0.50	0.80	0.94
2	0	1	2	resistance [MΩ]	0.55	2.87	4.48	5.33	5.68

(2.1) $$V_{\text{out}}=\frac{R_{Q}+R_{T_{Q}}}{R_{P}+R_{Q}+R_{T_{Q}}+R_{T_{P}}}\cdot V_{\text{logic}}\approx \frac{R_{Q}}{R_{Q}+R_{P}}\cdot V_{\text{logic}},$$

where $R_{T_{Q}}$ and $R_{T_{Q}}$ are the resistances of the transistors in the 1T1R arrays. The resistances of the transistors are negligible compared to the resistances of the memristive devices when the transistors are fully open. By representing the resistances of memristive devices in state 2, 1 and 0, in the form $R=a^{2}\cdot b$ , $a^{1}\cdot b$ and $a^{0}\cdot b$ , respectively, the division relationship of the inputs can be transformed into


(2.2) $$V_{\text{out}}=\frac{a^{Q}\cdot b}{a^{Q}\cdot b+a^{P}\cdot b}\cdot V_{\text{logic}}=\frac{1}{1+a^{P-Q}\cdot b}\cdot V_{\text{logic}},$$

where b is the base, a is the geometric factor, P and Q are the inputs. In this work, the inputs 0, 1 and 2 are mapped to $R=a^{0}\cdot b$ , $a^{1}\cdot b$ and $a^{2}\cdot b$ , respectively. By defining $V_{out}(q\geq p)=V_{out}(q=p)$ , meaning that, using $V_{out}$ in case of q=p to represent the group values of $V_{out}$ when q≥p , the IMP function can be implemented:


(2.3) $$V_{out}=\left\{ \begin{array}{l} \frac{1}{a^{0}+1}\cdot V_{logic}\;(Q\geq P)\\ \frac{1}{a^{P-Q}+1}\cdot V_{logic}\;\;(Q<P) \end{array}\right..$$

The simulation result is listed in table 1 (right-hand side) and all the $V_{out}\geq 0.5$ V are clustered into logic value 2. Naturally, the value of 0.5 V serves as a threshold in this case. In the subsequent sections, the experimental validation of this concept is described in detail.

## 3. Test environment

The experimental measurements were performed on an integrated ReRAM chip out of the process offered by the Circuits Multi-Projects manufacturer. The access transistors and digital select logic were implemented in the HCMOS9A 130 nm CMOS process from STMicroelectronics, while the memristive devices were deposited in a back-end-of-line process by CEA-LETI [27] with TiN/HfO_2_/Ti as material stack. In the custom layout, each ReRAM was connected in series with a common-source n-channel MOSFET as 1T1R configuration.

For the measurements, a 512×32 1T1R *crossbar array* is selected in the ‘Vertical 1T1R’ configuration, where the transistor gates are connected by the word lines (WL) 1–32, which are aligned in parallel with the bit lines (BL) 1–32 connecting the common ReRAM electrode. Orthogonal to these lines, the transistor sources are connected by the source lines (SL) 1–512. The array layout and the corresponding IMPLY configuration are depicted in figure 1*b*.

The ReRAM chip is mounted into a custom design *printed circuit board*, which is part of the measurement set-up. The rest of the set-up includes a *Labjack T7* data acquisition board for controlling the digital on-chip circuitry and an *external voltage supply* for $V_{DD}$ . The analogue voltage signals are applied via an *Aixacct systems µ C module*, which also provides the current measurement. The high-resolution voltage measurement is realized via a *PicoScope 5000a* PC oscilloscope. The *Labjack T7*, the *µ C* module as well as the PicoScope oscilloscope are connected to a PC for automated measurement control. The capability of this set-up for performing memristive 1T1R CIM operations have been demonstrated before [28,29].

Two distinct measurement operations are performed within this crossbar array set-up, the single device read-out for data access and the two-device IMPLY as logic operation. For reading the state of a single ReRAM device, a specific pair of SL and combined WL/BL are selected for applying a low-voltage read pulse. The foremost objective is to ensure that the device state remains undisturbed during the read-out.

The IMPLY operation is depicted in figure 1*a*. During the operation, two 1T1R neighbouring bitcells are selected which contain the devices P and Q . Neighbouring in this context means that both devices are connected to the same BL while having individual SL and WL connections. The device selection is achieved by applying a high gate voltage $V_{\text{Gate}}$ at the WL connected to of each 1T1R bitcell transistor gate. Then the transistor source of one cell is biased via its SL with the logic execution voltage, $V_{logic}$ , while the other transistor source is set to ground potential, GND. This resembles a voltage divider configuration with the shared BL being the middle node. Here, the voltage drop $V_{out}$ occurs. To measure $V_{out}$ as well as the input signal $V_{logic}$ , both signals are connected to separate oscilloscope channels. The bias schemes for both, single device read-out and two-device IMPLY are listed in table 2.

**Table 2.** Bias schemes for crossbar array measurements and programming.

operation	SL 1	SL 2	BL	WL
single-device read-out	$V_{\text{read}}=0.2\;V$	—	GND	$V_{\text{Gate},\;Read}=5\;V$
two-device IMPLY	$V_{\text{logic}}=0.3\;V$	GND	—	$V_{\text{Gate},\;IMPLY}=3\;V$
SET state 0	GND	—	$V_{\text{SET}}=2\;V$	$V_{\text{Gate},\;SET}=1\;V$
state 1	GND	—	$V_{\text{SET}\;1}=1\;V$	$V_{\text{Gate},\;SET\;1}=0.75\;V$
state 2	GND	—	$V_{\text{SET}\;2}=0.75\;V$	$V_{\text{Gate},\;SET\;2}=0.7\;V$
RESET	$V_{\text{RESET}}=1.5\;V$	—	GND	$V_{\text{Gate},\;RESET}=5\;V$

The programming of individual ReRAM devices within the crossbar array is achieved by applying electrical pulses along the corresponding WL, BL and SL. The first step in the multi-level programming is the device initialization into the high resistive state using first a SET and then a RESET pulse. Following this, either a regular SET (logic value 0) or reduced SET pulse with decreased gate voltage is applied to gradually shift the device state towards the low resistive state (LRS). The reduction in gate voltage is crucial as it allows a finer control over the resulting LRS state for the logic values 1 and 2. The according voltage schemes are also listed in table 2.

Similar to the common write/programming process in ReRAM devices [30,31], to further increase the precision of the multi-level programming, a program-verify algorithm is conducted afterwards, which consists of continuous loops of device programming and resistance verification. The device resistance state is evaluated by measuring the read-out current induced from a 200 ms pulse with an applied voltage of 0.2 V. If this measured device resistance does not match the targeted range for the specific state, an additional SET pulse, in case of resistance higher than the target, or RESET pulse, for resistances lower than the target, is applied to the device. With each iteration, the pulse amplitudes of SET and RESET are adjusted as the resistance of the ReRAM device narrows closer to the target value until the target resistance range is finally achieved. As a starting point, the pulses listed in table 2 are selected. In the SET direction, the BL voltage is reduced step-wise towards 0.7 V, while the RESET pulses are reduced in $V_{g,\text{RESET}}$ towards 2.0 V and in $V_{\text{RESET}}$ towards 1.0 V. As this procedure is conducted manually, the voltage decrement with each iteration is not optimized; despite this, the concept we proposed has still been successfully validated.

## 4. Results and discussion

In the validation process, the resistive states $R_{0}=5.1\text{k}\Omega$ , in the range (4.57– 5.49kΩ ), $R_{1}=19\;k\Omega$ in the range (16.6– 20.2kΩ ) and $R_{2}=60.3\;\text{k}\Omega$ in the range (57.7– 64.7kΩ ) are assigned to logical states 0, 1 and 2, respectively. In this case, the base *b* in equation (2.2) is selected as 5100 and the geometric factor *a* is set as approximately 3.7. Because of the programming limits of the chip, the geometric factor *a* is not ideal as $\frac{R_{2}}{R_{1}}=3.4$ , whereas $\frac{R_{1}}{R_{0}}=3.73$ . Nevertheless, this approach was successfully validated experimentally. Furthermore, the effect of the variability of the memristive devices is also investigated.

### (a) D2D variability

**Figure 2.**
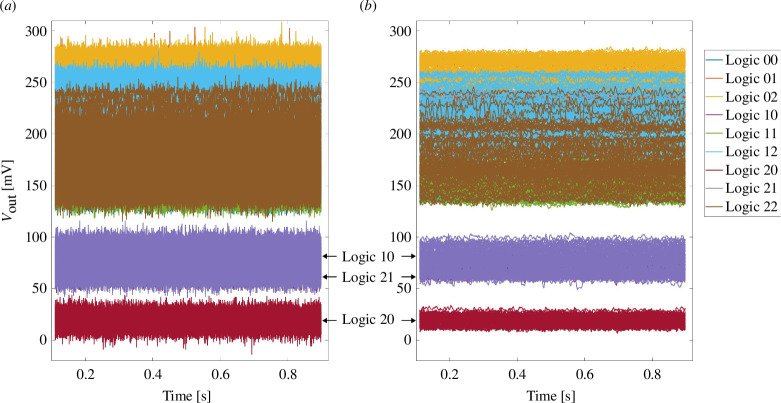
(*a*) Read out voltages and (*b*) noise reduced read out voltages in D2D tests. Logic XY means logic combination P=X and Q=Y .

In the D2D variability tests, 10 out of 30 devices (located in the same row of the 1T1R array) are programmed to $R_{0}$ , 10 devices are programmed to $R_{1}$ and 10 devices are programmed to $R_{2}$ , initially. For each logic computation test, two devices are selected and connected in serial form. The voltage at the middle node of the connection is read out and mapped to the logical output. There are 90 combinations for the case of equal logic inputs ( P=Q.P,Q∈(0,1,2) ), while 100 combinations for the case of unequal logic inputs ( P≠Q.P,Q∈(0,1,2) ). The read-out voltages are shown in figure 2*a*. To reduce the read noise in the test set-up, the signals are filtered with a moving average window of size 10. Moreover, to gather reliable results, the signals are cut out from approximately 0.1 to 0.9 s. (All the following signals are processed in the same method.)

As illustrated in figure 2*a*,*b*, the results of the logic input combination 20 are marked in red and are located at the bottom of the diagram, mapping the logical output 0. The results of the logic input combinations 21 and 10 are marked in dark purple and light purple and overlap, falling in the range of 50–100 mV, indicating the logical level 1. All results of the other input combinations overlap and yield read-out voltages above approximately 130 mV, corresponding to the cluster P≥Q mapped to logic value 2. Considering the noise reduced signals, there is a significant gap (approx. 20 mV) between the different output logical levels, thus, validating the ternary logic concept D2D tests. In the left-hand diagram, the separation between output logical values can still be discerned, although the peaks of different logical levels might overlap. Note that increasing the applied read-out voltage results in improved read margins but may also increase the probability of read-disturb faults.

**Figure 3.**
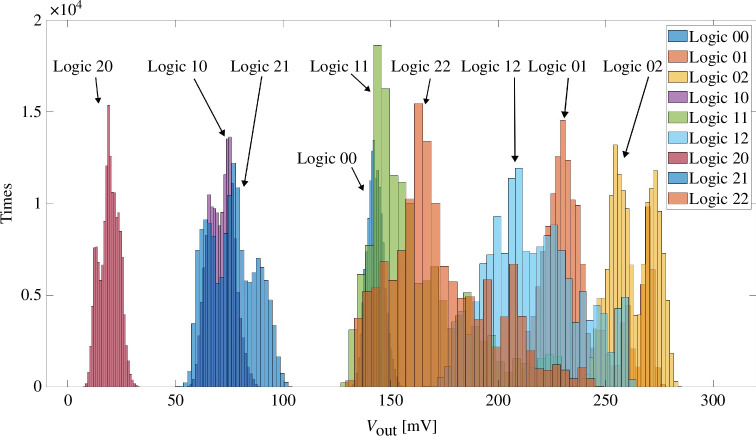
Histogram of the noise reduced read out voltages in D2D tests. Logic XY means logic combination P=X and Q=Y .

The processed signals are presented in the form of a histogram in figure 3. There are clear gaps between each output logic value. Note that, each logic combination has more than one peak in the diagram, indicating the D2D variability in the tests. Although the 10 devices are programmed to the same resistive states, they differ from each other. The smaller the resistance values, the narrower is the range of the read-out voltages and the fewer peaks they might have.

### (b) C2C variability

**Figure 4.**
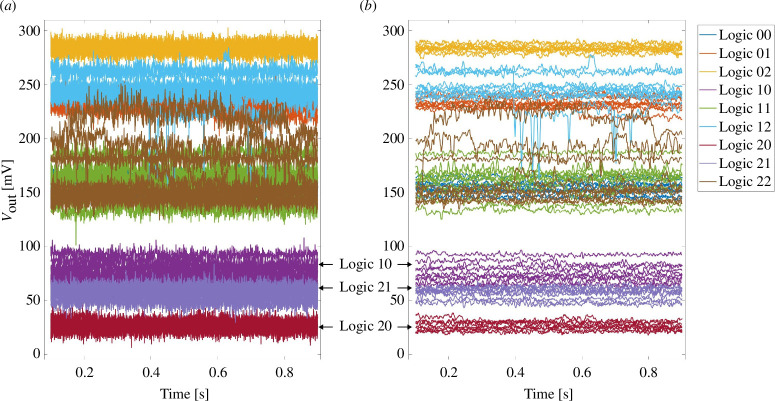
(*a*) Read out voltages and (*b*) noise reduced read-out voltages in C2C tests. Logic XY means logic combination P=X and Q=Y .

In C2C variability measurements, only two devices are used. In the single cycle test, the devices are programmed to the desired states and then connected in series. Subsequently, the voltage values of the intermediate nodes are read out. This ‘programming-and-read’ operation is executed 10 times for each logic combination. The final results are presented in figure 4.

Similar to the D2D test results, clear gaps between the read-out voltages mapped to different output logic values can be found. The gaps are located in a similar range as in the D2D tests, logic 0 in (17.6–36.5 mV), logic 1 in (43.1–96.8 mV) and logic 2 in (127.7–291.7 mV). It is readily noticeable from the graph that there are significant fluctuations in the signals of logic combinations 12 and 22, which may be attributed to random jumps of individual oxygen vacancies within the conducting filament [32]. Despite the small gap between logic values 0 and 1 in figure 4*a*, the validation of the logic functionality is achieved in the denoised read-out voltages as shown in figure 4*b*.

### (c) R2R variability

**Figure 5.**
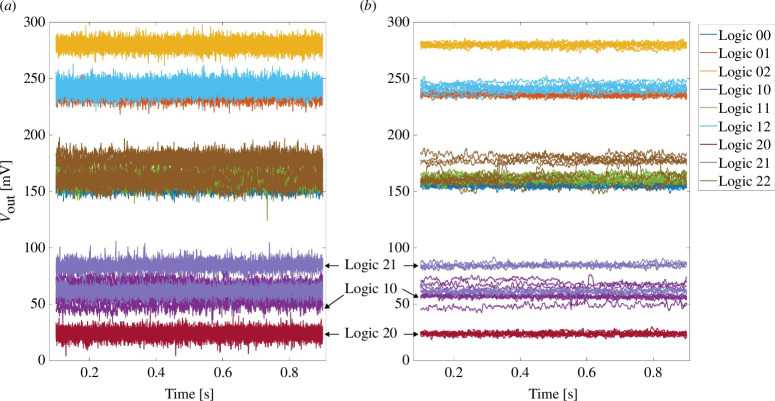
(*a*) Read out voltages and (*b*) noise reduced read out voltages in R2R tests. Logic XY means logic combination P=X and Q=Y .

In R2R variability measurements, two devices are also used. In the single-read test, the devices are programmed to the desired states and then connected in series. Then, the voltage values of the middle nodes are read out 10 times without reprogramming the devices, in contrast to the C2C tests. The test results are presented in figure 5. The gaps, not only between the different output logic values but also between the different logic combinations, clearly appear in the diagram. Thus, the concept is validated in this test, too. Even though the devices are only programmed once, the read-out voltages differ in different reading cycles, e.g. in the case of logic combination 21, there are two distinctly separated signal groups, which would indicate two main peaks in the histogram.

### (d) Parallel operation

**Figure 6.**
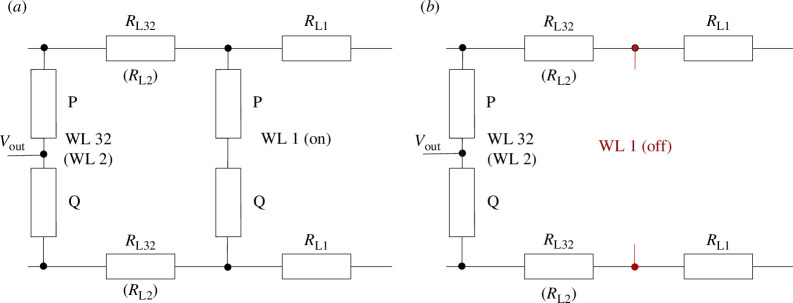
(*a*) The equivalent circuit diagram working in the parallel-operation mode with sets WL32 (WL2) and WL1. (*b*) The equivalent circuit diagram working in the single-operation mode with sets WL32 (WL2). All the resistances of select transistors are neglected under the assumption of $R_{LRS}\gg R_{Tran}$ . WL N means the two series connected devices on word line N.

**Figure 7.**
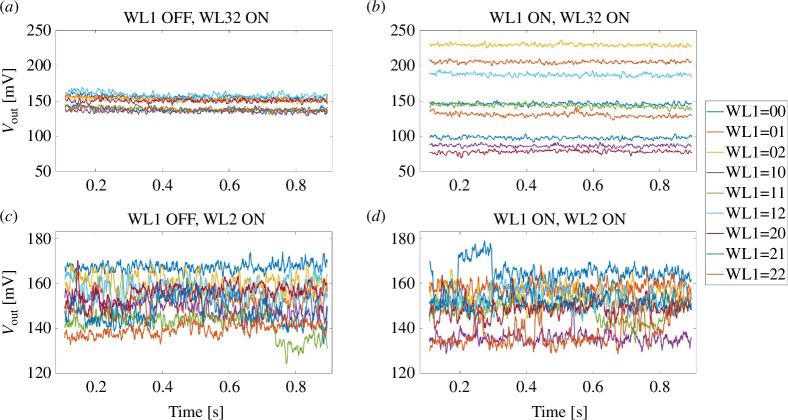
The noise reduced read out voltages for logic combination 11 on WL32 and WL2 in parallel-operation mode tests. (*a*) WL1 off, WL32 on. (*b*) WL1 on, WL32 on. (*c*) WL1 off, WL2 on. (*d*) WL1 on, WL2 on; ‘off’ means single-operation mode, ‘on’ means parallel-operation mode. WL1=XY means for the cases of logic combination on word line WL1 P=X and Q=Y .

All tests conducted in the preceding section belong to single-operation mode test, as in each test, only two devices are interconnected for logic computation. To take better advantage of the scale effect of the crossbar array, a parallel-operation mode of this concept is also investigated. In parallel-operation mode, two sets of logic calculations are performed simultaneously on the same SL. Each set of logic calculations is executed by two series of connected devices P and Q . Note, that only the vertical 1T1R crossbar array is suitable for the parallel-operation mode of this concept as the 1T1R structures share the SL in the row (column) and the BL in the column (row). Rows and columns are perpendicular to each other. The equivalent circuit diagrams (again neglecting the resistance of the transistors) of the parallel- and single-operation mode are shown in figure 6*a*,*b*, respectively. In fact, single-operation mode is a special case of parallel-operation mode with devices having an infinite resistance. As the core principle of this approach relies on the series-connected voltage division principle, the investigation of factors influencing voltage division on the devices is therefore a focal point of research. Thus, the wire resistances may have decisive effect in this scenario. To investigate its influence, we performed a set of comparative experiments by varying the wire resistance size. Furthermore, we designed another set of comparative experiments where we measured and analysed the output voltage at the middle node in both single-operation mode and parallel-operation mode configurations to study the effect of parallel-operation mode. As illustrated in figures 6 and 7, we conducted comparative experiments between WL1, WL32 and WL1, WL2 configurations to study the effect of line resistances. The parallel operation of WL1 and WL32 (WL2) should resemble the worst (best) case as the line resistance $R_{L32}$ ( $R_{L2}$ ) between the pairs is largest (smallest). In each experiment, we measured the voltage at the middle node of the two devices sharing word line WL32 (WL2). The logic computation on WL32 (WL2) should be executed twice, once with the two devices on WL1 working (parallel-operation mode) and once not (single-operation mode). For the parallel-operation mode, the select transistors in WL32 (WL2) and in WL1 are activated while all other select transistors are deactivated. For the single-operation model, only WL32 (WL2) is activated using the select transistors. As aforementioned, clear gaps between read-out voltages mapped to logic output 0, 1 and 2 (by P=Q ) should appear. The read-out voltages ranging from 0 to 180 mV are the most critical for logic computation as the gaps are expected in this region based on the previous measurement. To investigate exactly this range, six out of nine logic input combinations 00, 10, 11, 20, 21 and 22 are applied on WL32 and WL2. For each logic input combination in parallel-operation mode on WL32 (WL2), all nine logic combination on WL1 are tested. Considering the results shown in the D2D, C2C and R2R tests (single-operation mode) and the results shown in figure 7*a*,*c*, we can conclusively draw the inference that the line resistance itself is negligible compared to the resistances of the working devices, i.e., $2\cdot R_{LRS}\gg R_{L32}+R_{L1}$ . As illustrated in figure 7*a*,*b*, it is evident that the logic validation has failed in the parallel-operation mode, as the read-out voltages for the case WL32 = 11 range from 25 to 230 mV, although it is approximately 150 mV in the single-operation mode. In this experiment, the parallel-operation mode increases the current flowing through (cf. figure 6*a*), resulting in the decrement of the voltage drop on P and Q of WL1. The voltage drop on P and Q on WL32 (WL2) decreases further because two $R_{L32}$ ( $R_{L2}$ ) are serially connected to them (voltage division). Because of the nonlinearity and variability of the devices on WL1, disturbance will be amplified through the wire resistance and transmitted to WL32. The greater the difference between the two inputs of WL1, the greater is the resulting effect (cf. figure 7*b*). Moreover, WL32 itself also has the same issues. Thus, all these factors led to the validation failure. In figure 7*c*,*d*, however, the read-out voltages are more or less unaffected by the parallel operation. The only variable is the wire resistance $R_{L2}\ll R_{L32}$ . In this comparative experiment, the wire resistances are negligible compared with $2\cdot R_{LRS}$ because of the short distance between the devices on WL1 and the ones on WL2. The wire resistance in this case is too small to amplify and transfer the disturbance of WL1 to WL32.

We have reason to infer that as long as the wire resistance is sufficiently small compared to the resistances within the working devices, parallel-operation mode is feasible and promising. Thus, to leverage the parallel operation, the 1T1R array needs to be designed in a way that this condition is fulfilled.

## 5. Conclusion

In summary, we have experimentally validated a reading-based 3R ternary Łukasiewicz imply logic concept on a 1T1R array. The influence of D2D variability, C2C variability and R2R variability of memristive devices on gate functionality were analysed. C2C and D2D variability are more critical to logic computation as they reduce the read margin significantly. Furthermore, the potential of parallel operation is also discovered (proven) when the wire resistances are small enough compared to the sum of the resistances of the serial connected devices. To increase the read margin, research can be conducted in the following areas: seeking devices with smaller variability; appropriately adjusting the ratio of the resistive states, finely tuning it to a more suitable range, as the most ideal ratio in the case of linear symmetrical resistors is 2.89; programming the resistance states of the devices more accurately; exploring memristive devices with weaker nonlinearity and better symmetry. The interference of wire resistance on computation results, as demonstrated in the parallel-operation mode, limits the application of this concept in large-scale computational arrays. However, this issue might be mitigated by carefully selecting closely matched-operation pairs. For future work, investigating methods to reduce ReRAM variability and to enhance its reliability will be an important research direction. Additionally, exploring the feasibility and fault tolerance of this concept under different conditions is also a meaningful area of study. In final practical applications, issues such as circuit complexity, control of input signals, measurement of output voltage signals and optimization of the programming-verify algorithm are all challenges that need to be addressed in future work.

## Data Availability

We have done some tests, measured the voltages. Data can be accessed for now through my Onedrive link: [33].
